# How have sheep breeds differentiated from each other in Morocco? Genetic structure and geographical distribution patterns

**DOI:** 10.1186/s12711-021-00679-2

**Published:** 2021-11-04

**Authors:** Asmae Kandoussi, Bouabid Badaoui, Ismaïl Boujenane, Mohammed Piro, Daniel Petit

**Affiliations:** 1 Department of Animal Production and Biotechnology, Institut Agronomique et Vétérinaire Hassan II, Rabat-Instituts, PO Box 6202, 10101 Rabat, Morocco; 2 Laboratory of Biodiversity, Ecology and Genome, Mohammed V University, 4 Avenue Ibn Battouta, B.P. 1014 RP, Rabat, Morocco; 3 Department of Medicine, Surgery and Reproduction, Institut Agronomique et Vétérinaire Hassan II, Rabat-Instituts, PO Box 6202, 10101 Rabat, Morocco; 4 Glycosylation et Différenciation Cellulaire, EA 7500, Laboratoire Peirene, Université de Limoges, 123 av. A. Thomas, 87060 Limoges Cedex, France

## Abstract

**Background:**

Based on the relatively homogeneous origin of the sheep breeds in Morocco that originate mainly from Iberia, it is highly relevant to address the question of how these very diverse sheep populations differentiated from each other. The Mountains of the High Atlas and Middle Atlas are expected to constitute North–South and West–East geographical barriers, respectively, which could have shaped the history of the differentiation of sheep breeds. The aim of this study was to test this hypothesis by considering the genetic structure and the spatial distribution of five major breeds (Sardi, Timahdite, Beni Guil, Boujaad and D’man) and one minor breed (Blanche de Montagne), by analysing the mtDNA control region, using 30 individuals per breed.

**Results:**

Phylogenetic and network analyses did not indicate any clear separation among the studied breeds and discriminant component principal analysis showed some overlap between them, which indicates a common genetic background. The calculated pairwise F_ST_ values and Nei’s genetic distances revealed that most breeds showed a moderate genetic differentiation. The lowest and highest degrees of differentiation were retrieved in the Beni Guil and Boujaad breeds, respectively. Analysis of molecular variance (AMOVA) indicated that more than 95% of the genetic diversity occurs within individuals, while between- and within-population variabilities represent only 1.332% and 2.881%, respectively. Isolation-by-distance, spatial Principal Component Analysis (sPCA), and spatial AMOVA analyses evidenced clear examples of geographical structuration among the breeds, both between and within breeds. However, several enigmatic relationships remain, which suggest the occurrence of complex events leading to breed differentiation.

**Conclusions:**

The approaches used here resulted in a convergent view on the hypothetic events that could have led to the progressive differentiation between the Moroccan breeds. The major split seems to be linked to the West–East barrier of the Middle Atlas, whereas the influence of the High Atlas is less obvious and incompletely resolved. The study of additional breeds that have settled near the High Atlas should clarify the relationships between the breeds of the West part of the country, in spite of their small population size.

**Supplementary Information:**

The online version contains supplementary material available at 10.1186/s12711-021-00679-2.

## Background

Unraveling how the sheep breeds of a region have progressively differentiated from each other is a difficult task. The main reason is that countries are often situated on a route of migration and thus have experienced several independent migration events in the past, resulting in further admixture of the populations. This has been verified for example in Italy [1], Portugal [2], Spain [3], European countries [4], and Algeria [5]. Recently, mtDNA analysis revealed that 79% of the genetic background of Moroccan sheep derives from sheep from the Iberian Peninsula and 21% from sheep from a territory between Middle East and Africa, with a calculated expansion time ranging from 7100 to 8600 years B.P. and moderate exchanges in both directions in more recent times, which is coupled with an influence from Italian sheep [6]. It confirms the role of the Mediterranean Sea route on the genetic movements of livestock, between North Africa, Europe and Middle East [7–10]. Due to its geographical and historical position, Morocco seems to be a particularly suitable example to address how sheep breeds have originated.

Morocco is a sheep breeding country and with more than 0.5 sheep per inhabitant, it has the highest production potential of the Maghreb countries [11]. The number of sheep is estimated at around 19.9 million heads [12]. The local sheep breeds in Morocco constitute an important reservoir of genetic diversity, play a crucial economic and social role for the rural populations, and hold a ritual role in religious festivals and other socio-cultural traditions. They are well adapted to different agro-ecological zones over the whole country, and enhance fodder resources that are rather marginal in terms of quality as well as by-products of crop production [13]. Ninety-nine per cent of the Moroccan sheep are composed of the so-called “local” type, with 43% of the so-called “common” sheep and 57% of pure sheep. Among the latter, 40% have a well-defined standard and the remainder are mainly composed of the so-called Mountain sheep [14]. Six breeds, which are phenotypically well distinguished and adapted to different ecosystems, constitute the main sheep flock (all thin-tailed): Sardi, Timahdite, Beni Guil, D’man, Boujaad and Blanche de Montagne. However, several other endemic and foreign populations (including the Ouled Djellal breed from Algeria) are not yet characterized and do not benefit from conservation programs.

Based on historical information, the Moroccan sheep breeds are divided into three main populations. The first one is the Mountain population, previously known as Berber, i.e. the most ancient one in the country and constituted in part by the Blanche de Montagne breed. Many studies have shown that the Mountain sheep populations in Morocco are characterized by specific phenotypic traits and a good adaptation to their environment, which suggests a unique genetic diversity [15–17]. The second population is formed by breeds reared in the Eastern and Western Plateaus of the country. The Eastern Plateaus are mainly populated by the Beni Guil breed, while the Western ones are occupied by the Sardi breed, previously known as the "Beni Meskine type”, and the Boujaad breed or the ex “Tadla type”. Sagne [17] hypothesized that this population derived from the Mountain population with animals of increased size at low altitudes, while the original small Berber stock remains in the mountains. The third population includes the breeds found on the Atlantic coast, mainly represented by the Beni Ahsen population, now nearly extinct. Concerning the Timahdite breed, Boujenane and Ait Bihi [18] proposed that it results from crossbreeding between the “Tadla type” and the Mountain population. Finally, the D’man breed, which has settled in the palm groves of the southern oases of Morocco (regions of Draa and Tafilalet), has been promoted from the early 1970s onwards for its high prolificacy, but historical data of its origin are not available.

Besides these old historical considerations, it can be hypothesized that the mountains themselves have shaped the genetic structure of the sheep breeds. On the one hand, the High Atlas is a long chain that separates the Northern and Southern populations with few passes. On the other hand, the Middle Atlas constitutes another barrier that isolates the Western and Eastern populations. The aim of this study was to test these historical and geographical hypotheses in the differentiation of the Moroccan breeds. To reach this goal, our purpose was to provide new information about the genetic variability and structure of the main sheep breeds in Morocco, using mtDNA control region sequences, and to examine the pattern of their geographical distribution. The results are compared to other studies that investigated the diversity of sheep breeds in Morocco, in terms of morphological traits [19], blood proteins [20], and different genetic markers [6, 21–23].

## Methods

### Data analysis

The 191 sequences analyzed correspond to GenBank accession numbers MN229085 to MN229277 and represent the six main sheep breeds in Morocco: Sardi, Timahdite, Beni Guil, D’man, Boujaad and Blanche de Montagne. The mean number of individuals per breed was 32, ranging from 27 (D’man) to 37 (Timahdite). Between 13 and 17 flocks per breed were investigated. For each flock, the blood of one to three unrelated individuals based on their genealogy was collected. More details about the sampling are in Additional file 1: Table S1. In addition, we provide a map that illustrates the breeding areas and the sampling of the breeds (see Additional file 2: Fig. S1). The shared haplotypes between the breeds were defined by the DNAsp version 10 software [24] and were used to construct a phylogenetic tree using the MEGA software [25], based on 1000 bootstrap replicates. The phylogenetic tree was constructed using the maximum likelihood (ML) method. The best model according to the lowest Bayesian Information Criterion corresponded to the Tamura 3-parameter model [26], which included a Gamma distribution (5 discrete categories) and Invariant sites, (G + I). The individuals with each haplotype are represented by colored circles according to breeds, and the haplogroup of each individual is marked by a different shape.

The median-joining network was performed using the Network software 10.1.0.0 version [27]. The “Star Contraction” option was used to reduce the large dataset, and the “MP calculation” procedure according to the Neighbor-joining method was used to remove unnecessary vectors and median links and to avoid reticulations. Genetic diversity indices were calculated using the DNAsp software [24]. The significance of the values corresponding to each subpopulation was established by using the PAST program version 2.17c with a Student t-test for unequal variances [28].

Discriminant analysis of principal components (DAPC), implemented in the adegenet package R [29] was applied to a mtDNA dataset, to examine the genetic structure of the populations and to assess the degree to which breeds differ from each other when considering prior information on breeds. The DAPC approach is assumed to optimize the separation of individuals into predefined groups based on a discriminant function of principal components. Moreover, DAPC analysis was used to assign individuals and to obtain the membership probability, which presents the overall genetic background of an individual.

Evidence for population genetic structure was assessed using Analysis of MOlecular VAriance (AMOVA) that was conducted with the poppr.amova function as implemented in the poppr package [30]. Spatial analysis of the molecular variance was performed with the SAMOVA software [31], using the coordinates of the center of the traditional breeding area of each studied breed.

The pairwise F_ST_ values were calculated in order to analyze the degree of differentiation between the studied breeds. The F_ST_ values and their significances (p-values) were calculated between all combinations of breeds. Computation was performed with the hierstat package [32], using pairwise.neifst (for F_ST_ values) and boot.ppfst (for p-values) functions, while the overall F_ST_ value was calculated using the basic.stats function implemented in the same package. Nei’s genetic distances between the breeds were calculated using the dist.genpop function implemented in the adegenet package [33]. These distance values were considered to construct neighbor-joining trees using the PAST program version 2.17 [28]. The parenthetic forms were then exported to generate the tree with a radial option using the FigTree vers 1.4.3 software (http://tree.bio.ed.ac.uk/software/figtree/).

Spatial analysis of principal components (sPCA) was computed with the R software [34] in the adegenet package [33]. The coordinates used were those of the samples. Different connection networks were tested and a comparison of the results led us to choose as connection network the optimal number (K) of nearest neighbors (K = 10), which maximizes the clear separation of breeds. Spatial structures detected by sPCA were tested using the global and local permutation tests and Moran’s eigenvector maps [35]. The elevation map that is necessary for the next steps was produced using the Raster package [36]. In order to assess spatial patterns, the sPCA results were visualized on the elevation map by plotting the samples according to their geographical coordinates, and coloring them according to their respective scores along the first and second sPCA components. The graphical representation was obtained by using the adegenet package [33].

Isolation-by-distance (IBD) analysis was performed using the R. Mantel’s test in the adegenet package to check the correlation between genetic and geographical distances among populations with the mantel.randtest function (1000 permutations). To test whether this correlation results from a continuous or distant patchy cline of genetic differentiation, local densities of distances to disentangle both processes were plotted using a 2-dimensional kernel density estimation (function kde2d). The results were displayed using the MASS package [37]. The densities between points on a scatterplot can be used to determine whether the data originated from a single genetic cline or from two or more distinct regional clusters for which the test for IBD should be performed separately for each cluster.

## Results

### Haplotype analysis at the level of individual haplotypes

Among the 191 individuals that represent the six main sheep breeds in Morocco (Sardi, Timahdite, Beni Guil, D’man, Boujaad and Blanche de Montagne), 116 haplotypes based on the mtDNA control region were detected using DNAsp. The relationships between these haplotypes were examined by phylogenetic analysis. We constructed a consensus maximum likelihood tree of the 116 haplotypes (see Additional file 2: Fig. S2), which shows that the studied breeds are not differentiated in separate clusters and that many haplotypes are shared between more than two breeds. Haplotype 1 is the most shared haplotype, occurs in the six breeds and represents 14.7% of all samples.

**Fig. 1:**
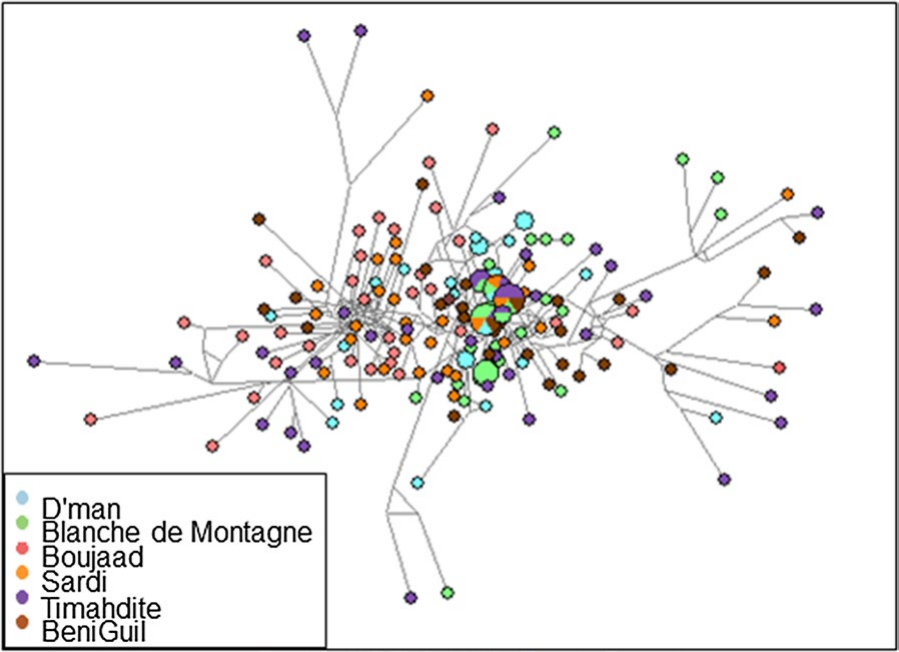
Network analysis. The figure was drawn using the Network software v.5.0.0.1, available on the website: http://www.fluxus-engineering.com/

The relationships between Moroccan sheep individuals were also evaluated through network analysis (Fig. 1). After running the star contraction option, the Network program revealed 167 different haplotypes, which is a much larger number than the above 116 haplotypes and is due to the difference between the algorithms in Network and DNAsp. In this analysis too, the breeds did not show a clear differentiation, except for the Boujaad breed which appeared somewhat separated from the other breeds at the periphery of the network and associated with some individuals of the Timahdite breed. The center of this network is star-like shaped, thus revealing no breed structure, with six haplotypes being shared between breeds.

### Genetic variability and genetic structure

**Fig. 2:**
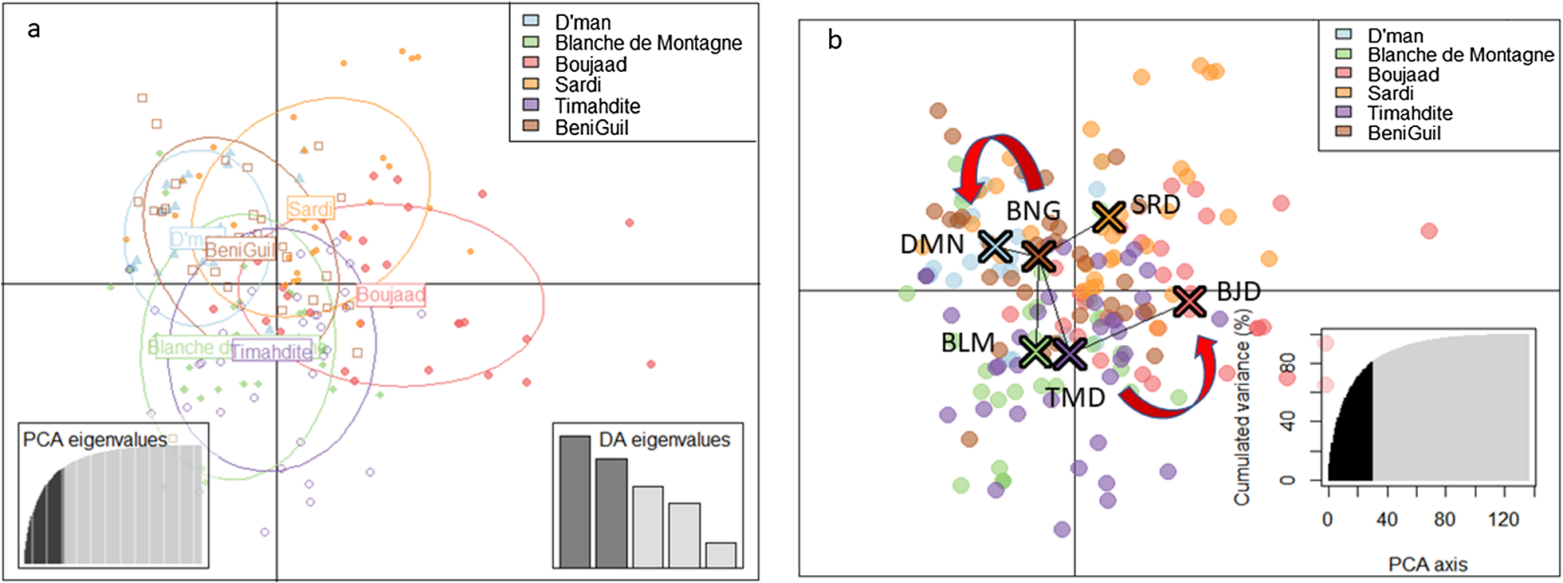
DACP analysis. **a** Distribution of individuals based on the first two discriminant functions. Individuals are represented by dots and groups are shown using 95% inertia ellipses depicted around individuals. The right hand side sub-shape represents the eigenvalues obtained from PCAc. **b** Discriminant analysis of principal components (DAPC) scatterplot. Dots represent individuals, with colors denoting the breed allocation to clusters. The percentages of cumulated variance explained by principal component 1 (PC1) to PC30 are shown in the bottom left corner of the figure. The minimum spanning tree based on the (squared) distances between clusters within the entire space is shown. *BNG* Beni Guil, *BLM* Blanche de Montagne, *BJD* Boujaad, *DMN* D’man, *SRD* Sardi, *TMD* Timahdite

A discriminant analysis of principal components (DAPC) was performed in order to infer the genetic structure of the breeds and their level of admixture. The clusters in DAPC were defined by a priori assumptions of population membership (K = 6). The number of retained principal components was defined using the α-score optimization proposed by Jombart and Collins [38], resulting in 30 PC retained as input for the discriminant analysis, and cumulatively explaining about 80% of the total of genetic variability. Visualization of the population structure based on the DAPC revealed a substantial overlap between the breeds (Fig. 2a), which was clearer in the first discriminant function (see Additional file 2: Fig. S3). In spite of this overlap, Fig. 2a reveals two pairs of sister breeds, one including D’man and Beni Guil and the other Timahdite and Blanche de Montagne. As for Boujaad, it seems to be the most distant breed from all other breeds (Fig. 2b). Moreover, the first discriminant function scatterplot (see Additional file 2: Fig. S3) shows that density varies along this axis, with D’man having the highest within-population density between its individuals (60%) and Boujaad the lowest (25%), while the other breeds have a medium within-population density (about 35 to 45%).

**Fig. 3:**
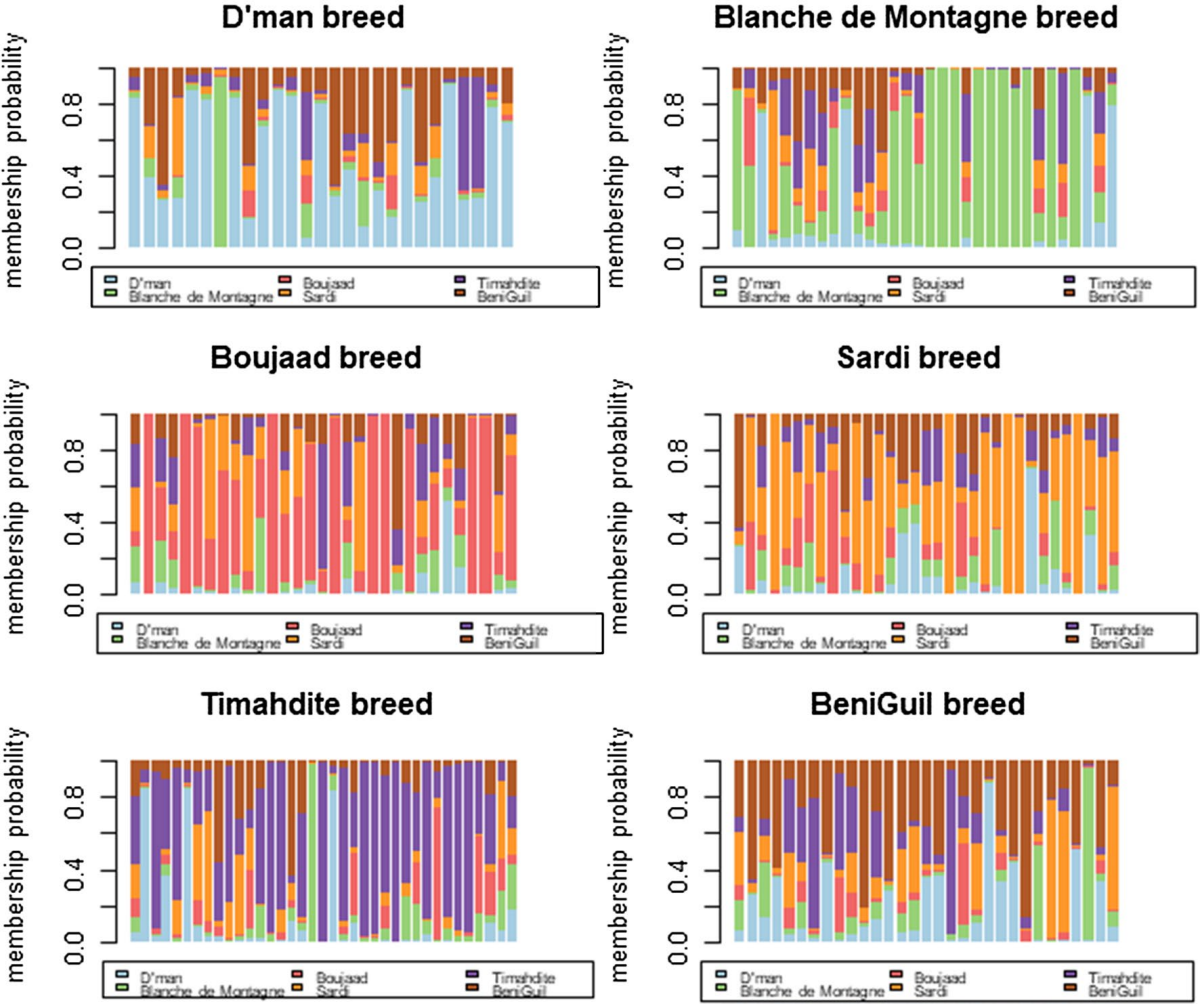
Probability of membership for each individual in the six studied breeds. Each column is representative of one individual and each breed is shown by a separate color

Figure 3 shows the results of the DAPC analysis with the prior assignment of groups and in the bottom plot a stacked bar plot of membership probability, which uncovered an average assignment probability of 56%. The highest and lowest population assignments were for the Sardi (63.63%) and Beni Guil breeds (45.16%), respectively, with intermediate assignment probabilities for the Timahdite (59.46%), Boujaad (58.06%), D’man (55.56%) and Blanche de Montagne breeds (53.12%).

We calculated the pairwise F_ST_ and pairwise Nei’s genetic distances (D) to explore the genetic relationships among the breeds (Table 1). Most of the F_ST_ values were significantly different from 0 (p < 0.001), except for the Beni Guil-D’man and Beni Guil-Sardi pairs (p > 0.1). Indeed, the Beni Guil breed is characterized by the smallest mean F_ST_ value at 2.71% and the Boujaad breed by the largest value at 8.08%. According to pairwise F_ST_ estimates, most breed pairs showed low to moderate genetic differentiation. The overall F_ST_ value was 0.042 (4.2%, p < 0.05). Most of the F_ST_ values were significant and the highest F_ST_ value was observed between the Boujaad and D’man breeds, while the lowest F_ST_ values (non-significant) were observed between the Beni Guil and Sardi and between the Beni Guil and D’man breeds. The genetic distance matrix results were analogous to those reported by the pairwise F_ST_ values (Table 1). To indicate the position of the root in the tree constructed based on these distances, we used the Portuguese breed Churra Algarvia as outgroup (see Additional file 2: Fig. S4). This figure shows that the Boujaad breed is situated at the base of the Moroccan breeds, with Timahdite, Blanche de Montagne and Sardi, and the Beni Guil-D’man pair of breeds successively branching off.

**Table 1:** Pairwise genetic differentiation between breeds

Breed	D’man	Blanche de Montagne	Boujaad	Sardi	Timahdite	Beni Guil	Churra Algarvia
D’man	–	0.007147	0.014738	0.007173	0.006537	0.003274	0.013700
Blanche de Montagne	0.0607***	–	0.012197	0.004843	0.004322	0.003980	0.00646
Boujaad	0.1072***	0.0809***	–	0.012779	0.011389	0.011443	0.014192
Sardi	0.0510***	0.0233***	0.0723***	–	0.004921	0.003597	0.006789
Timahdite	0.0502***	0.0219***	0.0687***	0.0226***	–	0.004123	0.006929
Beni Guil	0.0112 NS	0.0195***	0.0749***	0.0097 NS	0.0202***	–	0.007107
Churra Algarvia	0.1030***	0.0284***	0.0553***	0.0278***	0.0348***	0.0545***	–

Values above the diagonal represent Nei’s genetic distances (D) and values below the diagonal represent F_ST_ pair-wise values with significance: *p < 0.05; **p < 0.01; and ***p < 0.001

We performed AMOVA to quantify the genetic variation at three hierarchical levels: (i) among breeds, (ii) between individuals within breeds, (iii) and within individuals. The AMOVA results indicated that the genetic variance could be attributed almost entirely to individuals (95.787%), with low between- and within-population variabilities (1.332 and 2.881%, respectively) (see Additional file 3: Table S3). However, there was a significant difference in the molecular variance between breeds (p < 5%). The AMOVA analysis was also conducted at four hierarchical levels: (i) between groups, (ii) between breeds within groups, (iii) between individuals within breeds, (iv) and within individuals. Five breed groupings were tested, in order to consider a geographical genetic partitioning of the breeds. The first breed grouping took a northern group formed by the Sardi, Boujaad, Timahdite and Beni Guil breeds and a southern group formed by the D’man and Blanche de Montagne breeds into account. The second breed grouping was constructed to determine whether the chain of the Atlas Mountains constituted a geographical barrier, and thus the group of Sardi, Boujaad, Timahdite breeds was compared to the group of Beni Guil, D’man and Blanche de Montagne breeds. The third breed grouping was composed of the Beni Guil and D’man breeds against the remaining breeds, which could be isolated from the former by the North–South Mountains of Rif and Middle Atlas. The fourth breed grouping was conducted to test the result obtained from the pairwise F_ST_ values (see Additional file 2: Fig. S4), with the following three groups: (i) Sardi, Timahdite, and Blanche de Montagne; (ii) Beni Guil and D’man; and (iii) Boujaad. The fifth breed grouping was organized according to the most significant result from the SAMOVA analysis (see “Spatial structuration of genetic variation” section) as follows: (i) Sardi; (ii) Blanche de Montagne; (iii) Boujaad and Timahdite; and (iv) Beni Guil and D’man. The F_CT_ values associated with variation between groups were low and not significantly different from 0 for the first, second and fourth breed groupings, i.e. 0.15, 0.6, and 0.6%, respectively (p > 0.1), but they were significant for the third and fifth breed groupings (1.63%, p = 0.01 and 2.35%, p = 0.03, respectively) (see Additional file 3: Table S4).

In order to gain a deeper insight into the genetic variation within the breeds, we calculated the nucleotide diversity (π) and haplotype diversity (H_d_) for the different subpopulations of Sardi, Timahdite and D’man breeds. In the case of the Sardi breed, we compared the subpopulation of the Beni Meskine area (North-West) with the populations of the El Brouj and Kelaa Sraghna regions (South-East). For the Timahdite breed, we tested its subpopulations from the Timahdite (Middle Atlas) and Ghoualem areas (littoral plain), and for the D’man breed, we tested its subpopulations from the Draa-Dades (West) and Tafilalet areas (East). The p-values associated to the H_d_ estimates were not significant for the Sardi subpopulations, but significant at 5% and 0.1% for the D’man and Timahdite subpopulations, respectively. We took the π estimates into account since their p-values were at the level of 0.01% for the three breeds concerned. The results showed that the Sardi subpopulations of El Brouj and Kelaa Sraghna had a higher diversity than the subpopulation from the Beni Meskine area. Furthermore, the Timahdite subpopulation of the Ghoualem area appeared to be significantly more diverse than that from the Timahdite area, and the D’man subpopulation of Tafilalet had a significant higher diversity than that from the Draa-Dades area (see Additional file 3: Table S5).

### Spatial structuration of genetic variation

**Fig. 4:**
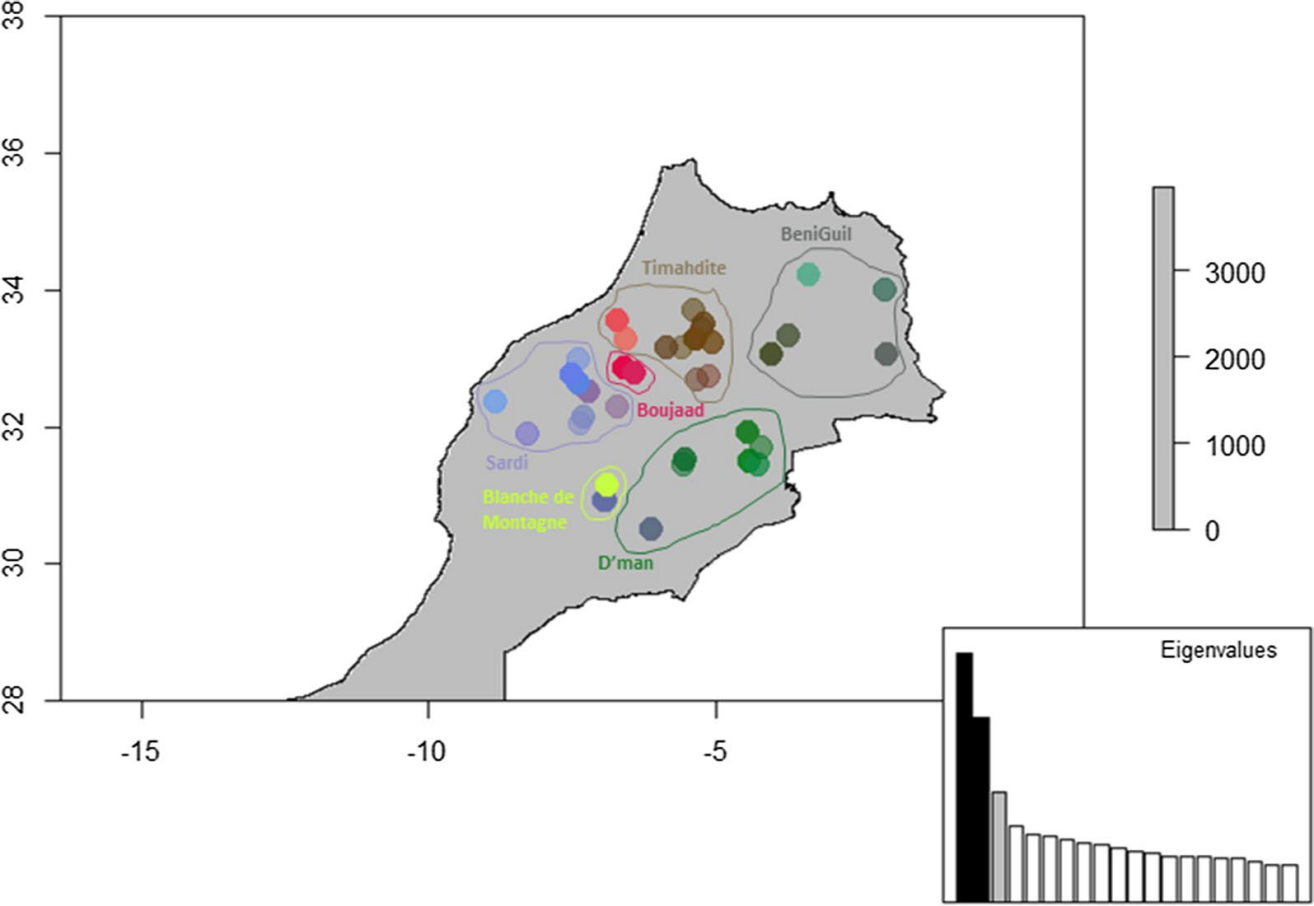
Spatial genetic structure detected by sPCA for six breeds from Northern Morocco. The figure represents a color plot of the first three principal components (PC) of the sPCA of Moroccan sheep data. Each dot corresponds to the sampled breeds (see the map of sampling distribution, in Additional file 2: Fig. S1). Each principal component is recoded as intensities of a given color channel of the RGB system: red (first PC), green (second PC), and blue (third PC). These channels are mixed to form colors representing the genetic proximities between the population individuals. The inset indicates the eigenvalues of the analysis, with color channels used to represent PC indicated on the corresponding eigenvalues

The patterns of the geographical distribution of genetic structures were investigated using spatial principal components analysis (sPCA). sPCA eigenvalues for each global and local structures are represented in Additional file 2: Fig. S5a. The analysis was carried out according to the K nearest neighbors (K = 10) connection network (see Additional file 2: Fig. S5b). The plot showing the spatial and variance components of eigenvalues revealed three eigenvalues that were clearly differentiated from the rest (noted λ1, λ3 and λ126 on Additional file 2: Fig. S5c with variances of 0.4, 0.47 and 0.68, and I-values of 0.4, 0.2 and -0.1, respectively, and that suggested potential genetic structuration at the geographical scale. The first three sPC represented only 38.1% of the total variation. The global Monte Carlo test (10,000 iterations) indicated no significant global and local spatial structures (p = 0.873 and p = 0.344, respectively). The eigenvectors of the three first global scores were plotted according to the geographical coordinates with a color gradient (Fig. 4). According to the first component (in red), the Boujaad breed population is close to the Ghoualem subpopulation of the Timahdite breed, whereas the Timahdite subpopulation of the Middle-Atlas region seems to be linked to the western Beni Guil subpopulations (brown dots). According to the second component (in green), two breeds, Beni Guil in the east and D’man in the south-central part, form a group. The Blanche de Montagne breed in the South West is split into two genetically distinct subpopulations: i.e. the isolated subpopulation in the Ghassat locality (yellow dot) and the subpopulation in the Iminlaoune locality (blueish dot), which seems to be close to the western populations of the D’man breed and to a lesser extent to the Sardi breed (third component, in blue).

**Fig. 5:**
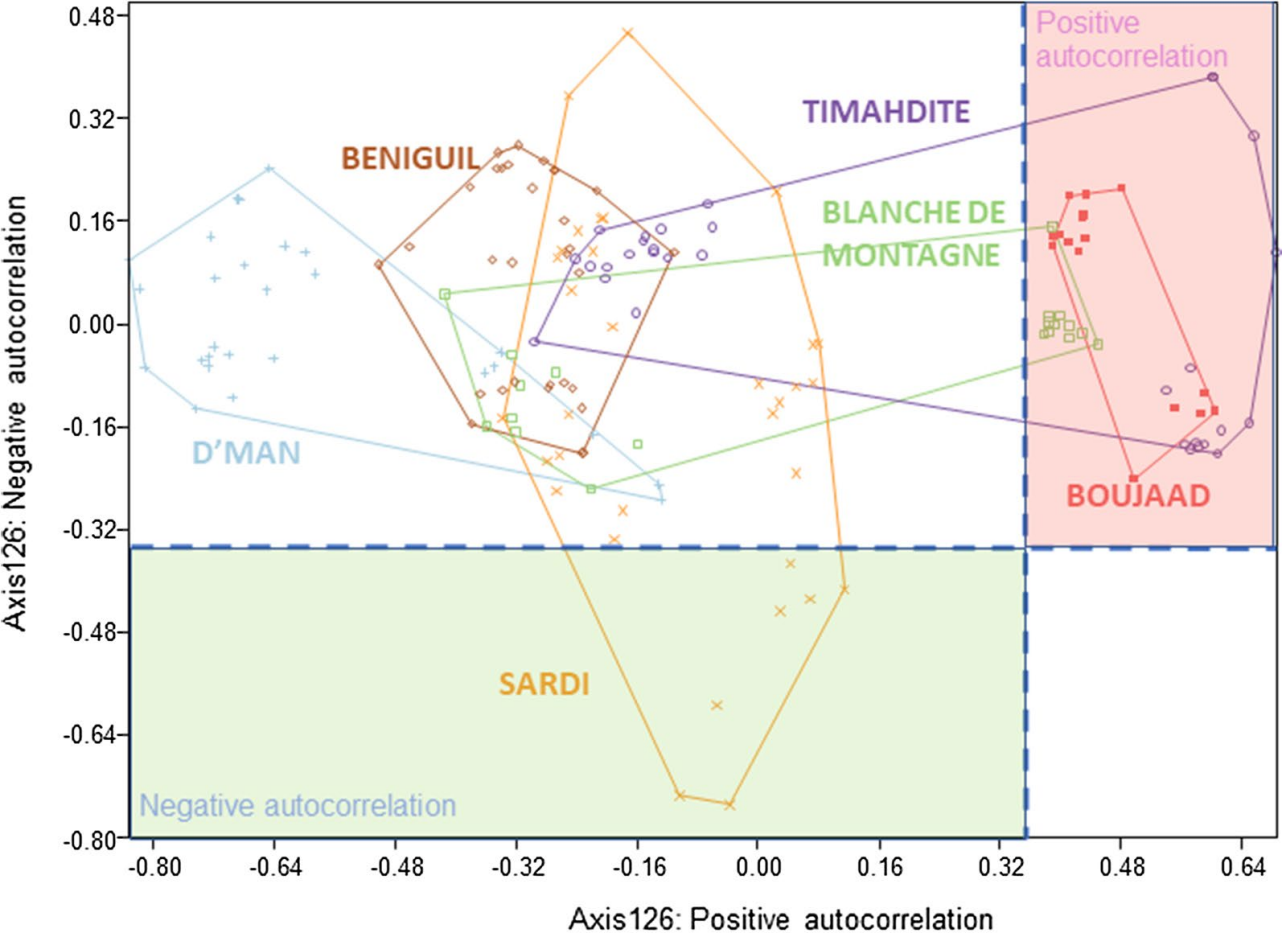
Spatial genetic autocorrelation scores (Axes 1 and 126)

In order to substantiate the geographical structuration of the breeds, the first two autocorrelation values (λ1, and λ126) of the sPCA were plotted onto a bidimensional plot (Fig. 5). The results show that there are four categories of breeds: (i) the Boujaad breed, which appears to be entirely positively correlated; (ii) the Timahdite and Blanche de Montagne breeds, which are separated into two parts, one that is positively autocorrelated and one that has a neutral autocorrelation; (iii) the Sardi breed, with individuals being divided into two groups, one that is negatively autocorrelated and one that has a neutral autocorrelation; and (iv) a category that includes the D’man and Beni Guil breeds, which have entirely neutral autocorrelations.

The spatial pattern was also assessed using the isolation-by-distance (IBD) approach. The Mantel test over the whole dataset of the six breeds was not statistically significant (r = − 0.0346, p = 0.759) and rejected the hypothesis of the geographical effect on a global genetic differentiation. The Mantel regression plot (see Additional file 2: Fig. S6) displayed two density nuclei without discontinuities, which suggest the presence of two genetic clusters that seem to be genetically distinct. Therefore, the IBD analysis was conducted on each breed, separately. It appears that the IBD pattern was verified in the case of the Blanche de Montagne breed and marginally for the Boujaad breed. The density plot of these breeds indicated two distinct scatters, and the test showed a slight correlation between the genetic and the geographical matrix with r = 0.148, p = 0.05 and r = 0.232, p = 0.06, respectively. The remaining breeds did not reveal any IBD patterns (see Additional file 2: Fig. S7).

Simultaneously, the spatial structuration of the genetic variation was explored by SAMOVA analysis. The number of clusters (K value) ranged from 2 to 5. At K = 2, the first breed to be separated from the others was Blanche de Montagne with a non-significant F_CT_ value. At K = 3, the second breed to be separated was Sardi with an F_CT_ p-value barely near to significance. At K = 4, the Boujaad and Timahdite breeds were organized in one group, the Beni Guil and D’man in another one, and the Blanche de Montagne and Sardi in two separate groups. The F_CT_ p-value for this K number was significant. Finally, at K = 5, the last breed to be differentiated was Boujaad, with a marginally significant F_CT_ p-value (Table 2).

**Table 2:** F_CT_ values for different numbers of population groups (K) inferred by the SAMOVA algorithm

K	Population grouping	F_CT_	p-values
2	(Blanche de Montagne) (D’man, Boujaad, Timahdite, Sardi, Beni Guil)	0.01689	0.17
3	(Blanche de Montagne) (Sardi) (D’man, Boujaad, Timahdite, Beni Guil)	0.01948	0.07
4	(Blanche de Montagne) (Sardi) (Boujaad, Timahdite) (D’man, Beni Guil)	0.02478	0.02
5	Blanche de Montagne) (Sardi) (Boujaad) (Timahdite), (D’man, Beni Guil)	0.03620	0.06

## Discussion

To better understand the history of the emergence of sheep breeds in Morocco, we investigated the genetic variability and structure of the six main Moroccan sheep breeds, by analyzing the mtDNA control region. Our results provide original information about the intra- and inter-genetic population structure and the geographical patterns of these breeds.

The AMOVA results revealed that the greatest part of the genetic diversity occurred among individuals (95.79%), while the variation between breeds and among individuals within breeds contributed only 1.33% and 2.88%, respectively. Although the variation between breeds was small it was significant and it confirms previously reported results on Moroccan breeds [23] and Tunisian breeds [39] based on the analysis of a microsatellite dataset. The overall differentiation between the Moroccan breeds, which was evaluated by the mean F_ST_ value, was 0.042, which is significantly different from 0 and although it is low, it is consistent with the historical gene flow across the country [21]. Still, this value remains higher than the F_ST_ values reported by several authors on North African sheep breeds based on microsatellite data, except for Egyptian breeds [40]. Gaouar et al. [23] reported an F_ST_ value of 0.036 for the five major sheep breeds of Morocco, and of 0.038 for six Algerian breeds [41]. Concerning the Tunisian breeds, the genetic differentiation was even lower, with F_ST_ values of about 1.7% for a set of four breeds [39, 42], and 0.021 for a set of six breeds, including one crossed population [43]. In contrast, higher values were reported for Central and South African [44, 45], European [46–51], and Oriental breeds [52, 53]. The lack of a clear genetic differentiation between the North African sheep breeds can be due to different reasons. On the one hand, North African has known multiple migration events of domestic animals from different regions and different civilizations, as well as large waves of agricultural commercial trades across the Mediterranean basin in the past [5, 10]. These elements have led to a large amount of genetic exchanges among domestic animals from these regions and North Africa. The result of these migration events outstands in the homogeneous genetic structure that is observed for these sheep populations of this area. On the other hand, the weak genetic structuration can reflect a low level of human selection pressure on the breeds.

The stacked bar plot of membership probability demonstrated that the Beni Guil breed had the lowest probability assignment, while the Sardi had the highest one. This large difference could result from differences in flock homogeneity. Several reasons may explain the low genetic differentiation of the Beni Guil breed. Until the 1960s, eastern Morocco was the center of the exportation of Beni Guil lambs to the European markets through Algeria under the “Petit oranais” label because they are highly appreciated for their organoleptic meat [16]. As a result, it can be predicted that this breed was affected by gene flow due to the commercial sheep activity during Morocco’s colonial period, a view that is supported by Belabdi et al. [21]. In addition, Kandoussi et al. [6] demonstrated that Beni Guil is the breed with the largest influence from European sheep (about 27% of foreign European connections). Furthermore, the Beni Guil breed faces a real danger of probable genetic dilution and flock decline due to the introduction of the Ouled-Djellal sheep breed from Algeria. Brisebarre [54] reported that about 200,000 heads of the Ouled-Djellal breed are introduced to Morocco each year (according to PDPEO [55]), a number that some breeders find largely underestimated, who cite an annual number of 500,000 sheep. The coexistence of these two breeds in the same geographical area is one of the main concerns in sheep farming in eastern Morocco. The Ouled-Djellal breed is actually not recognized by the Moroccan authorities and does not benefit from the genetic improvement program supervised by ANOC (Association Nationale Ovine et Caprine) [13]. Consequently, it was not possible to include a sampling of this breed in the current study and to evaluate its genetic introgression within the Beni Guil breed.

The pattern of the geographical distribution and genetic structuration for these sheep breeds was investigated by several approaches, which provided some elements highlighting the history of the differentiation of Moroccan sheep breeds. First, the distribution of the sequences throughout the phylogenetic tree demonstrated that the haplotypes present in each breed did not show a specific clade of breeds. This ascertainment could be a sign of ancient exchanges of genetic material between the breeds in the past. Moreover, the haplotypes are positioned on the tree with generally medium and long branches, which reflect an ancient origin of the diversity and suggest an ancient signature of sheep population expansion [56]. In addition, the star-like shape of the network analysis proves that the sheep populations have a robust genetic structure, meaning that individuals show a low level of relatedness, and thus suggest a low level of inbreeding. These findings are in agreement with the results of the DAPC approach. The scatter plot shows that the breeds overlap substantially, which reflects that part of the genetic background is shared between the breeds.

Second, the global test of isolation-by-distance, evaluated by the correlation between geographical and genetic matrices, was not significant, which refutes the idea of a progressive geographical structuration of the genetic diversity. This view is supported by the sPCA analysis which indicated no significant global and local spatial structures. However, the SAMOVA approach provided some interesting information, i.e. that the differences between breeds are significantly maximized when there are four groups, that there are two clusters each represented by two breeds, D’man and Beni Guil on the one hand, and Boujaad and Timahdite on the other hand, and that the other two breeds, Sardi and Blanche de Montagne, seem to be isolated.

This structuration of breed affinities is somewhat supported by the data provided by the F_ST_ values, the results of the AMOVA and the minimum spanning tree of DACP, which all pointed out the proximity between the D’man and Beni Guil breeds. This view is strengthened by the results of Gaouar et al. [23] based on microsatellite data. Concerning the Boujaad-Timahdite pair revealed by SAMOVA, it is in agreement with the minimum spanning tree of DACP and is consistent with the highest gene flow reported by Gaouar et al. [23] between these two breeds. However, the position of the Boujaad breed was challenged by the F_ST_ values, since it showed the highest degree of differentiation among the six tested Moroccan breeds, resulting in a position close to the Portuguese outgroup in the F_ST_-based tree. In the same way, the Network analysis revealed that the Boujaad individuals are the most distinctly separated from the other breeds. In fact, the Boujaad breed represents only 1.42% of the total sheep population in the country [57], and is reared in a narrow geographical area. In addition, the Boujaad breed has only very recently been promoted to the large public, which may explain its distinct genetic identity and its probable genetic drift compared to the other breeds. Finally, the very strong genetic influence from foreign animals, especially from the Middle East [6], could explain its special situation.

In summary, the strongest association concerns the Beni Guil and D’man breeds and to a lesser degree the Boujaad and Timahdite breeds. The geographical distribution is clearly related with these two identified pairs of breeds. Beni Guil and D’man are present in Eastern Morocco, while Timahdite and Boujaad are settled in the North-Center of the country. As a result, the main split revealed here could be the East–West axis of the mountains represented by the Middle Atlas. Within these two blocks, there are highly significant climatic boundaries between the members of these two pairs of breeds, as shown by Petit and Boujenane [58]. With regard to the Sardi breed, the reason of its enigmatic affinity remains to be explored. Indeed, the geographical continuity between Boujaad and Sardi is not reflected by our genetic data. The Blanche de Montagne breed also appears to be genetically distinct, which is certainly due to its isolated geographical rearing conditions on the southern flanks of the Atlas [59]. In spite of the proximity between this breed and the D’man breed, no clear genetic association is detected. These facts might explain the lack of a global genetic structuration revealed by the spatial autocorrelation approach. As the results show, the correlation between the genetic and geographical distances is not verified in many instances.

**Fig. 6:**
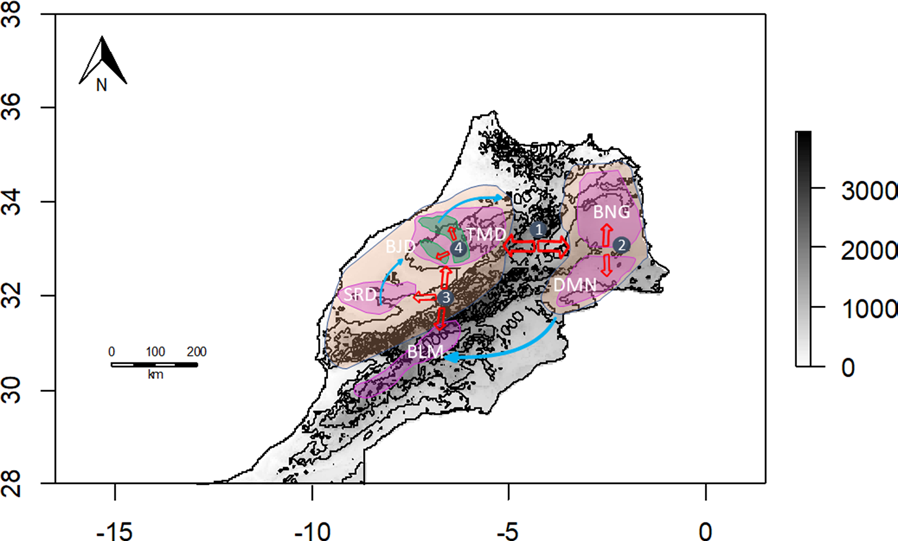
Summary of the hypothetic series of events leading to the main Moroccan breeds. The red arrows correspond to the successive events of breed separation. Note that the third event is not resolved. The blue arrows correspond to the extension of the populations subsequent to their differentiation, deduced from intra-breed differences in genetic variability indices (see Additional file 3: Table S5). *BNG* Beni Guil, *BLM* Blanche de Montagne, *BJD* Boujaad, *DMN* D’man, *SRD* Sardi, *TMD* Timahdite

At a finer scale, i.e. at the scale of the breeds, it may be questioned whether there is a geographical structuration. In a first attempt, a significant relationship was found between the genetic and geographical distances for the Boujaad and Blanche de Montagne breeds, which suggest a low level of genetic exchanges within their breeding areas. In contrast, no significant isolation-by-distance was evidenced for the Sardi, Timahdite, Beni Guil and D’man breeds, which thus reflects a higher gene flow between flocks within these breeds. Using the more precise method of autocorrelation developed by Jombard and Collins [38], significant differences were identified for the D’man, Sardi and Timahdite breeds. Since the D’man population of Tafilalet has a higher diversity value than that of Draa-Dades, it can be hypothesized that the early ancestors of this breed settled first in the eastern part of Morocco. This view supports the hypothesis that the D’man and Beni Guil breeds came from an Eastern ancestral population. With regard to Sardi, the same idea can be put forward, since the coastal populations of El Brouj and Kelaa Sraghna are more diversified than those of Beni Meskine, which suggest a southern origin for this breed. Concerning the Timahdite breed, the coastal population of Ghoualem showed higher π values than that of the Timahdite locality in the Middle Atlas, which argues in favor of a conquest of the Plateaus from the lower lands. The genetic differences within the breeds are confirmed using morphological measurements. Boujenane and Petit [19] showed that there are differences in morphological measurements between individuals of the same breed from different regions, which suggests the possible existence of sub-breeds or varieties. These latter data are preliminary but interesting, because it could help to understand the process of the historical settlement of the different breeds. A tentative synthesis of the hypothetic series of events that have led to the different populations of Moroccan breeds is provided in Fig. 6 and [(see Additional file 2: Fig. S7) for an overlook]. However, this hypothetic scheme needs to be tested at a more appropriate scale, and exploiting information contained in the whole mitochondrial and Y-chromosome genomes.

The weak geographical distribution of the genetic diversity found in the current study is consistent with previous studies on sheep breeds of Algeria using microsatellite data [60]. In addition, using single nucleotide polymorphisms, Belabdi et al. [21] revealed a high level of homogenization, such that some breeds with different origins appeared to be genetically undistinguishable. In addition, Ben Jemaa et al. [22] reported a pattern of multiple hybridization events within all North African sheep populations. Several investigations of the genetic structuration of goat breeds in North Africa resulted in similar results. Ouchene-Khelifi et al. [61] revealed the existence of a clear global genetic homogeneity concerning both Algerian and Moroccan stocks. Indeed, there was almost no genetic structuration among the Arabia (from Algeria), Draa, Black and Nord (from Morocco) breeds. Benjelloun et al. [62] attributed the weak differentiation of the Moroccan goat breeds to a high gene flow in parallel with the multiple migration waves along Northern Africa and the Mediterranean sea. The hypothesis of a gene flow between the North African and European flocks was also confirmed for goats. Finally, an analysis that combined mtDNA and chromosome Y loci data [63] reported a common origin for goat patrilines in both Mediterranean coastal regions, and detected traces of gene flow between the goat populations from Maghreb and the Iberian Peninsula, which corroborates evidence of past cultural and commercial contacts across the Strait of Gibraltar.

## Conclusions

To decipher the differentiation of sheep breeds within a country, it is necessary to combine several approaches, including the study of ancient DNA and the analysis of the genetic diversity and structure of the current populations. Due to a strong homogeneity of their genetic background, the sheep breeds of Morocco represent a favorable example, and the present work investigated the geographical structure of the breeds based on mtDNA sequences. Our findings show a good level of intra-breed genetic diversity and a low differentiation between breeds. Concerning the historical information about the origins of the different studied breeds, some elements are doubtful: there is no obvious opposition between the Mountain and Plateau breeds. Instead, we found a contrast between the Eastern breeds (D’man and Beni Guil) compared to the Western breeds, which may be in relation with the North–South barrier of the Rif and Middle Atlas. With regard to the Blanche de Montagne breed, it seems that the Anti-Atlas is a barrier that led to its isolation. Nevertheless, this breed does not seem to have any connection with the D’man breed, in spite of their geographical proximity. However, it would be useful to investigate other Mountain breeds to gain further insight into their genetic relationships with the Western Plateaus breeds. Moreover, a significant effort to enhance the number of samples of the Blanche de Montagne could increase our knowledge about the within-breed variation. Such improvements could help to better understand the situation of the Sardi breed. An interesting issue would be to consider the analysis of ancient DNA collected at a large scale to unravel the evolutionary events that have shaped the genetic structure of the current breeds. For example, there is a deep gap between the phenotypic traits of the D’man and Beni Guil breeds and gaining knowledge on the underlying process by which the D’man population originated is necessary.

## Supplementary Information

**Additional file 1: Table S1.** Details on the sampling of individuals (locality, access number of sequences, coordinates, flock number, haplogroups). **Table S2.** General statistics on the diversity indices of the whole dataset.

**Additional file 2: Fig. S1.** Sample map distribution, with the breeding areas of the breeds. **Fig. S2.** Phylogenetic tree of the haplotypes of Moroccan sheep breeds. The figure was drawn using the maximum likelihood method implemented in MEGA X [25], with the option Tamura 3-parameter model [26], including a Gamma distribution (5 discrete categories) and Invariant sites (G + I). **Fig. S3.** Density plot of breed individuals based on the first discriminant function. **Fig. S4.** Unrooted neighbour-joining tree based on pairwise F_ST_ values. Churra Algarvia was used as outgroup. The figure was drawn using the FigTree version 1.4.3 software available at: http://tree.bio.ed.ac.uk/software/figtree/. **Fig. S5.** sPCA analysis eigenvalues and network connection. The figures were drawn using the R software [34] and the adegenet package [33]: (**a**) A variant of the plot of sPCA eigenvalues; (**b**) Decomposition of sPCA eigenvalues; the dotted segments indicate on the abscissa the maximum variance of a principal component, and in ordinate the minimum and maximum values of Moran's I; and (**c**) Connection network between individuals of the sheep breeds. **Fig. S6.** Scatter plot generated based on genetic distances (Fst values) and geographical distances between Moroccan sheep breeds. The figure was drawn using the R software [34] and the adegenet package [33]. **Fig. S7.** Scatter plot generated based on genetic distances (F_ST_ values) and geographical distances for each sheep breed. The figure was drawn using the R software [34] and the adegenet package [33].

**Additional file 3: Table S3.** Analysis of molecular variance (AMOVA) in the six Moroccan sheep breeds. df: degree of freedom; SSD: sum of squares; MSD: mean squared deviations. **Table S4.** AMOVA results with different breed groupings. **Table S5.** Genetic diversity indices of subpopulations of the D’man, Sardi, and Timahdite breeds.

## Data Availability

This work was based on the analysis of the 191 sequences deposited in GenBank (MN229085-MN229277) obtained in our previous paper [6].
