# Supplementary material for: How have sheep breeds differentiated from each other in Morocco? Genetic structure and geographical distribution patterns
Source: Genet Sel Evol. 2021 Nov 4;53:83. doi: 10.1186/s12711-021-00679-2 (PMC8567669; doi:10.1186/s12711-021-00679-2)
Supplement: Supplementary file 2 — Additional file 2: Fig. S1. Sample map distribution, with the breeding areas of the breeds. Fig. S2. Phylogenetic tree of the haplotypes of Moroccan sheep breeds. The figure was drawn using the maximum likelihood method implemented in MEGA X [25], with the option Tamura 3-parameter model [26], including a Gamma distribution (5 discrete categories) and Invariant sites (G + I). Fig. S3. Density plot of breed individuals based on the first discriminant function. Fig. S4. Unrooted neighbour-joining tree based on pairwise FST values. Churra Algarvia was used as outgroup. The figure was drawn using the FigTree version 1.4.3 software available at: http://tree.bio.ed.ac.uk/software/figtree/. Fig. S5. sPCA analysis eigenvalues and network connection. The figures were drawn using the R software [34] and the adegenet package [33]: (a) A variant of the plot of sPCA eigenvalues; (b) Decomposition of sPCA eigenvalues; the dotted segments indicate on the abscissa the maximum variance of a principal component, and in ordinate the minimum and maximum values of Moran's I; and (c) Connection network between individuals of the sheep breeds. Fig. S6. Scatter plot generated based on genetic distances (Fst values) and geographical distances between Moroccan sheep breeds. The figure was drawn using the R software [34] and the adegenet package [33]. Fig. S7. Scatter plot generated based on genetic distances (FST values) and geographical distances for each sheep breed. The figure was drawn using the R software [34] and the adegenet package [33]. [file 12711_2021_679_MOESM2_ESM.pptx]

## Slide 1
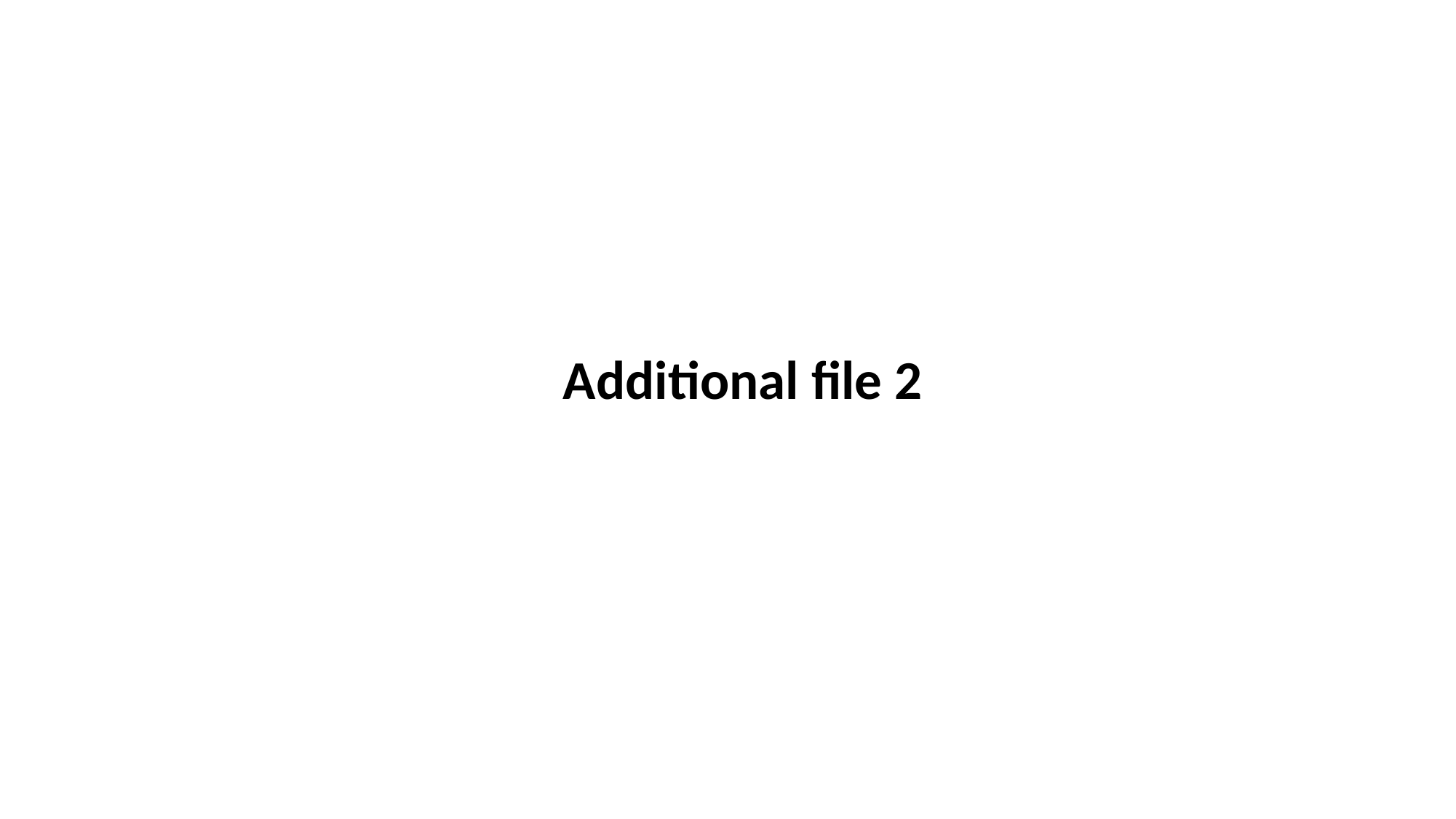

Additional file 2

## Slide 2
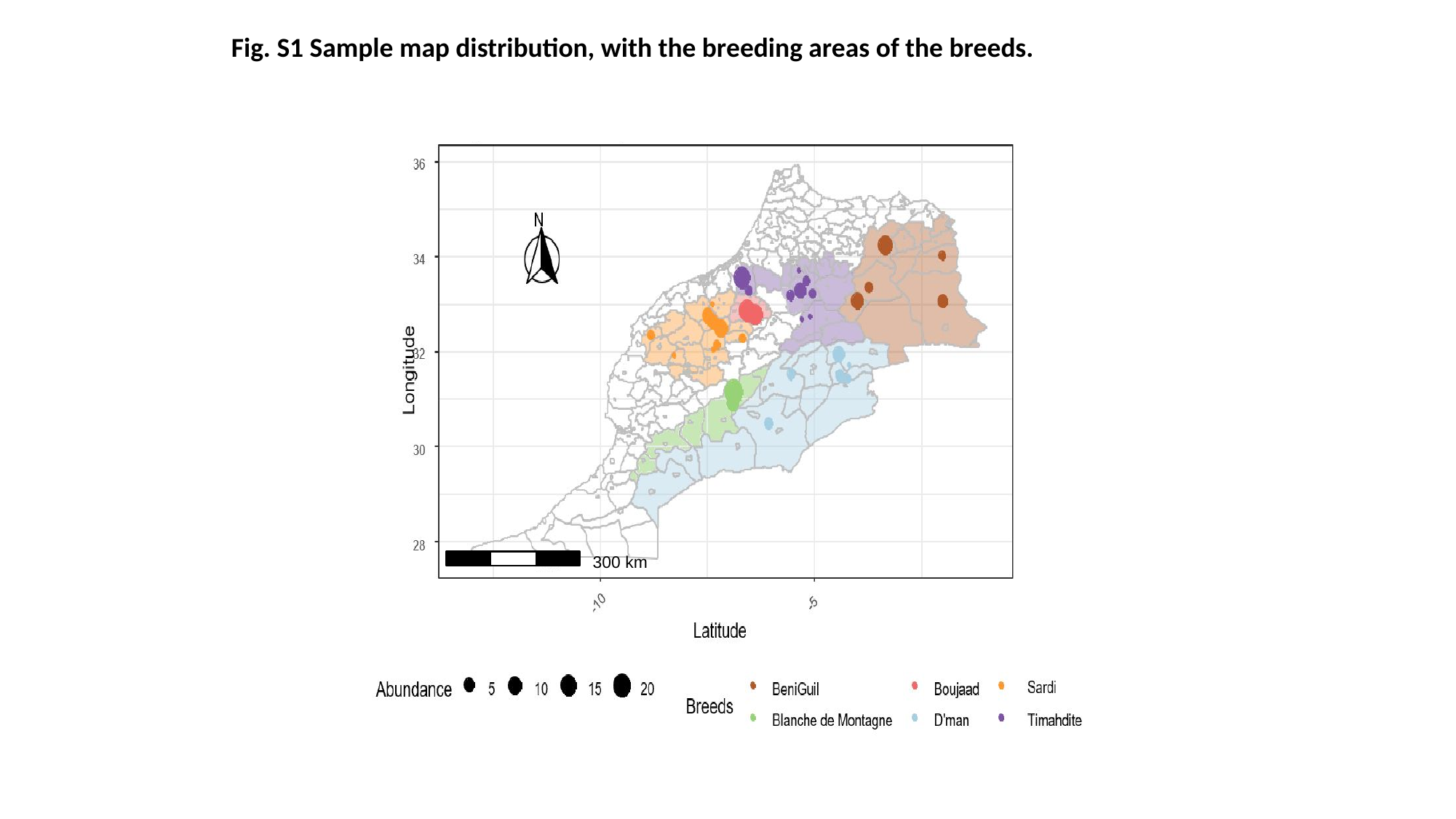

Fig. S1 Sample map distribution, with the breeding areas of the breeds.
300 km

## Slide 3
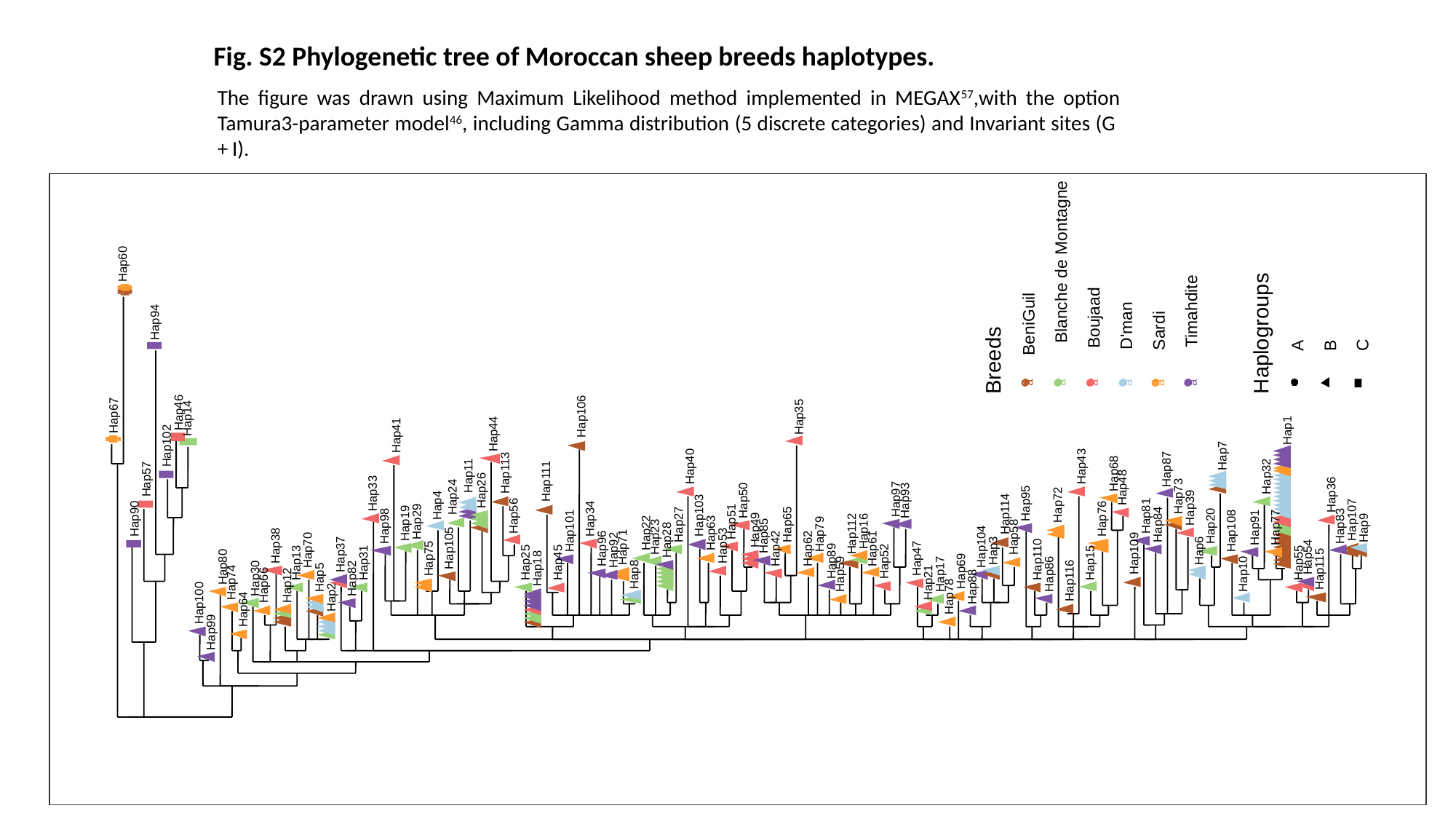

Hap102
Hap14
Hap100
Hap30
Hap38
Hap12
Hap13
Hap2
Hap37
Hap31
Hap33
Hap19
Hap29
Hap4
Hap105
Hap24
Hap11
Hap26
Hap113
Hap25
Hap18
Hap111
Hap101
Hap106
Hap34
Hap22
Hap23
Hap28
Hap27
Hap103
Hap35
Hap112
Hap16
Hap21
Hap17
Hap104
Hap3
Hap114
Hap110
Hap116
Hap15
Hap109
Hap39
Hap20
Hap108
Hap10
Hap32
Hap1
Hap115
Hap36
Hap107
Hap67
Hap60
Hap90
Hap57
Hap94
Hap46
Hap99
Hap80
Hap74
Hap64
Hap66
Hap70
Hap5
Hap82
Hap98
Hap41
Hap75
Hap44
Hap56
Hap45
Hap96
Hap92
Hap71
Hap8
Hap40
Hap63
Hap53
Hap51
Hap50
Hap49
Hap85
Hap42
Hap65
Hap62
Hap79
Hap89
Hap59
Hap61
Hap52
Hap97
Hap93
Hap47
Hap78
Hap69
Hap88
Hap58
Hap95
Hap86
Hap72
Hap43
Hap76
Hap68
Hap48
Hap81
Hap84
Hap87
Hap73
Hap6
Hap7
Hap91
Hap77
Hap55
Hap54
Hap83
Hap9
Fig. S2 Phylogenetic tree of Moroccan sheep breeds haplotypes.
The figure was drawn using Maximum Likelihood method implemented in MEGAX57,with the option Tamura3-parameter model46, including Gamma distribution (5 discrete categories) and Invariant sites (G + I).
Blanche de Montagne
Timahdite
Boujaad
BeniGuil
D'man
Haplogroups
Sardi
A
B
C
Breeds
a
a
a
a
a
a

## Slide 4
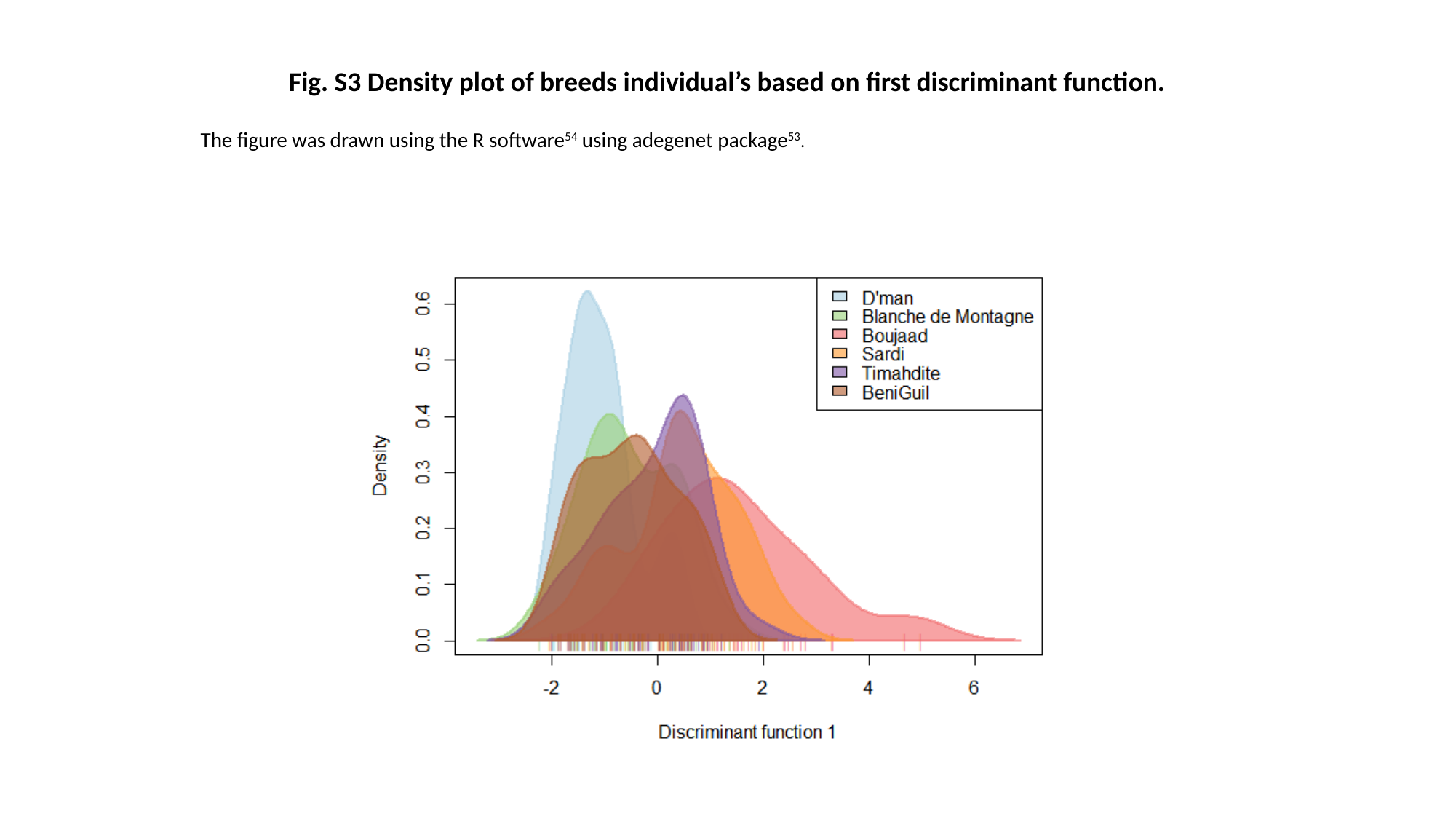

Fig. S3 Density plot of breeds individual’s based on first discriminant function.
The figure was drawn using the R software54 using adegenet package53.

## Slide 5
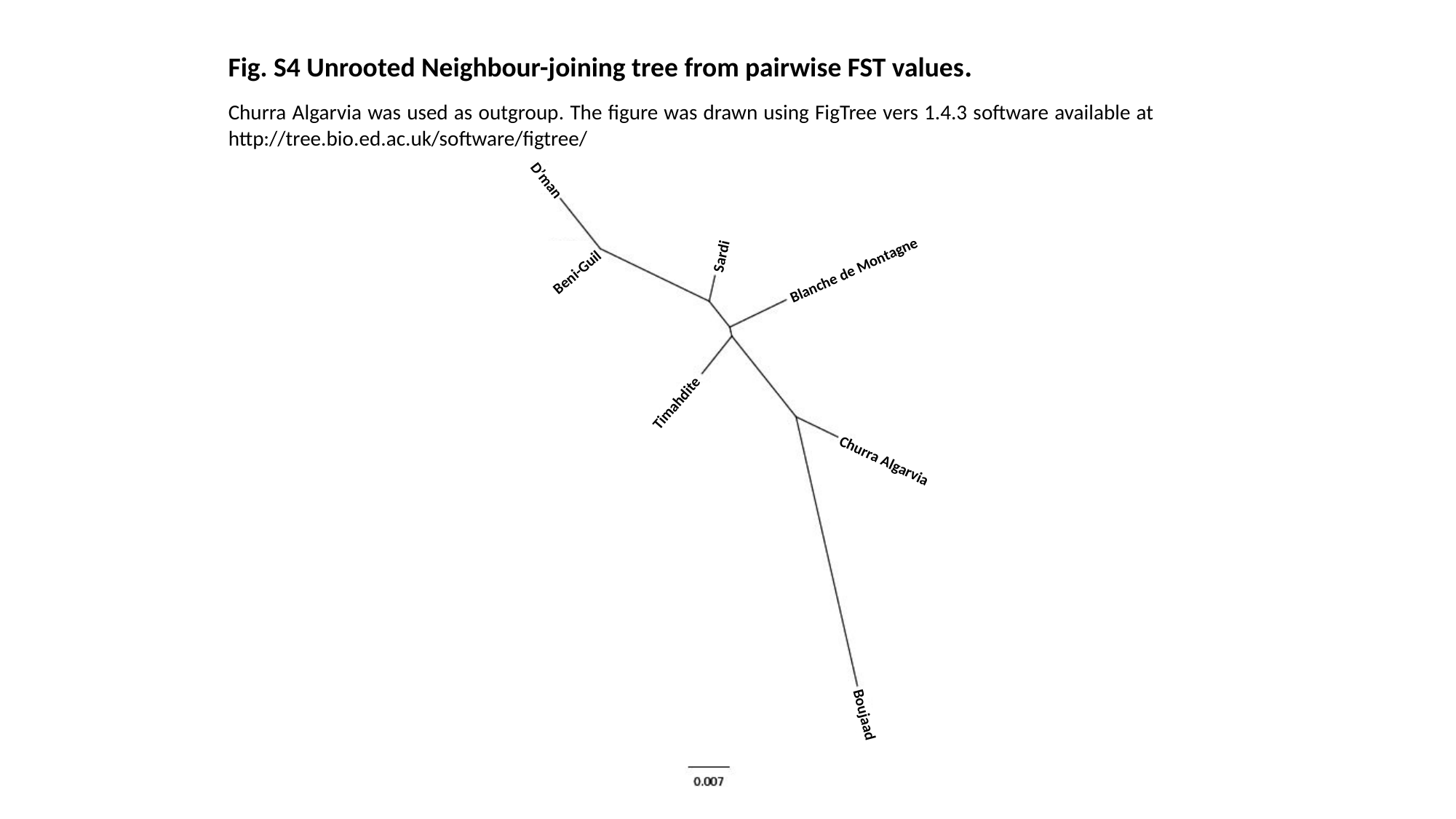

Fig. S4 Unrooted Neighbour-joining tree from pairwise FST values.
Churra Algarvia was used as outgroup. The figure was drawn using FigTree vers 1.4.3 software available at http://tree.bio.ed.ac.uk/software/figtree/
D’man
Sardi
Beni-Guil
Blanche de Montagne
Timahdite
Churra Algarvia
Boujaad

## Slide 6
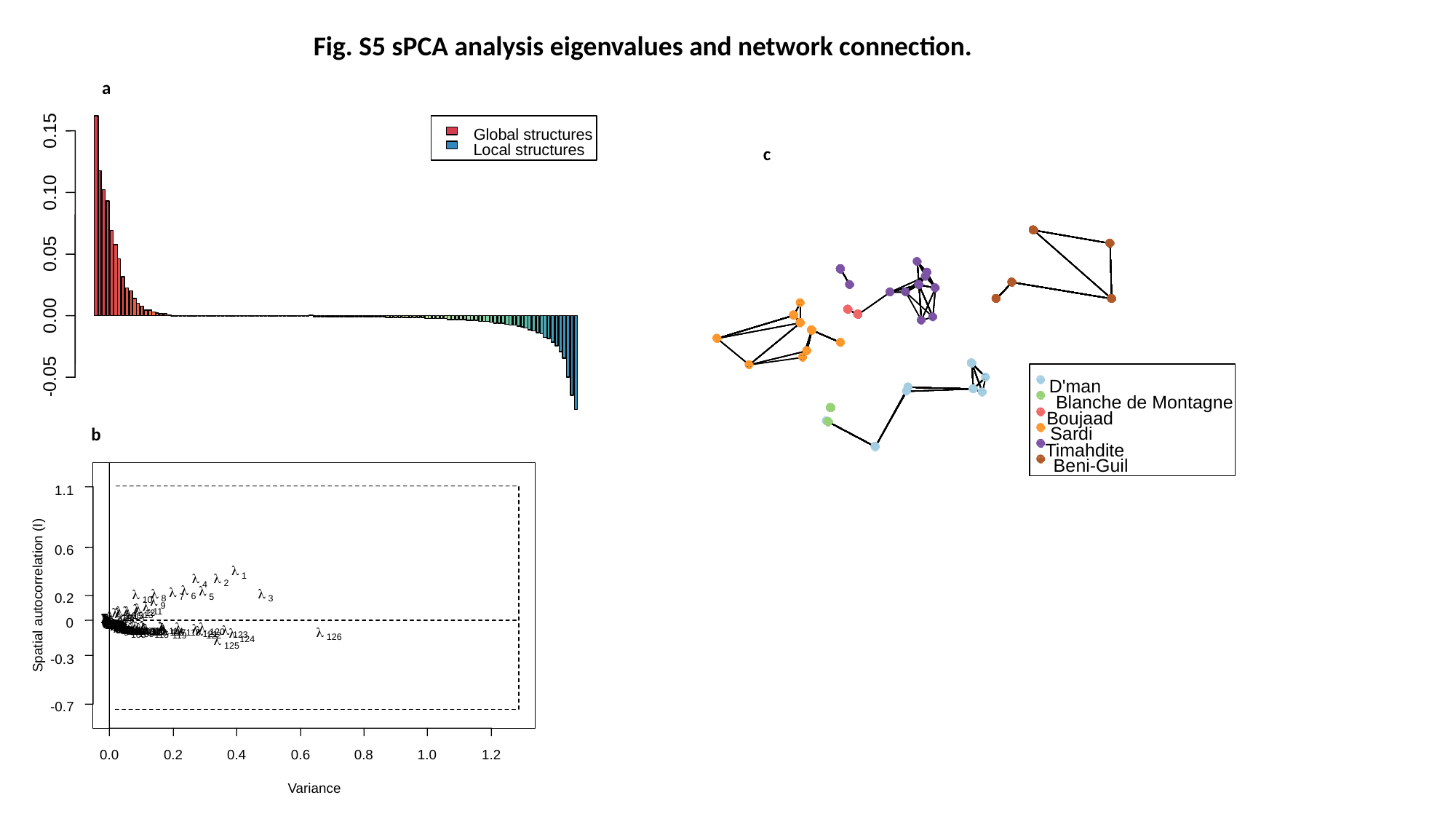

Fig. S5 sPCA analysis eigenvalues and network connection.
a
0.15
Global structures
Local structures
0.10
0.05
0.00
-0.05
c
D'man
Blanche de Montagne
Boujaad
Sardi
Timahdite
Beni-Guil
b
1.1
0.6



1
2
4





Spatial autocorrelation (I)

0.2
6
7
5

8
3
10


9







11

12

13














16
15




14










19
17








18
0














20















21








22










26
30
24
31
33
25
27
35
23
28
34
32
29
36








37
38
39
41
42





40
43
44
45
47
50



46
48
51
53


49
52
54
55
58
57
59
60
61
63
66

56
62
64
69
71
74
67
70
72
73
75
76
77
81
82
80
83

65
68
78
79
85
86
87
89
90
95

84
88
91
92
93
97
98
106
108
110
114
96
100
99
101
102
103
104
107
111
120
94
112
113
116
117
118
109
121
105
115
123
119
122

126
124
125
-0.3
-0.7
0.0
0.2
0.4
0.6
0.8
1.0
1.2
Variance

## Slide 7
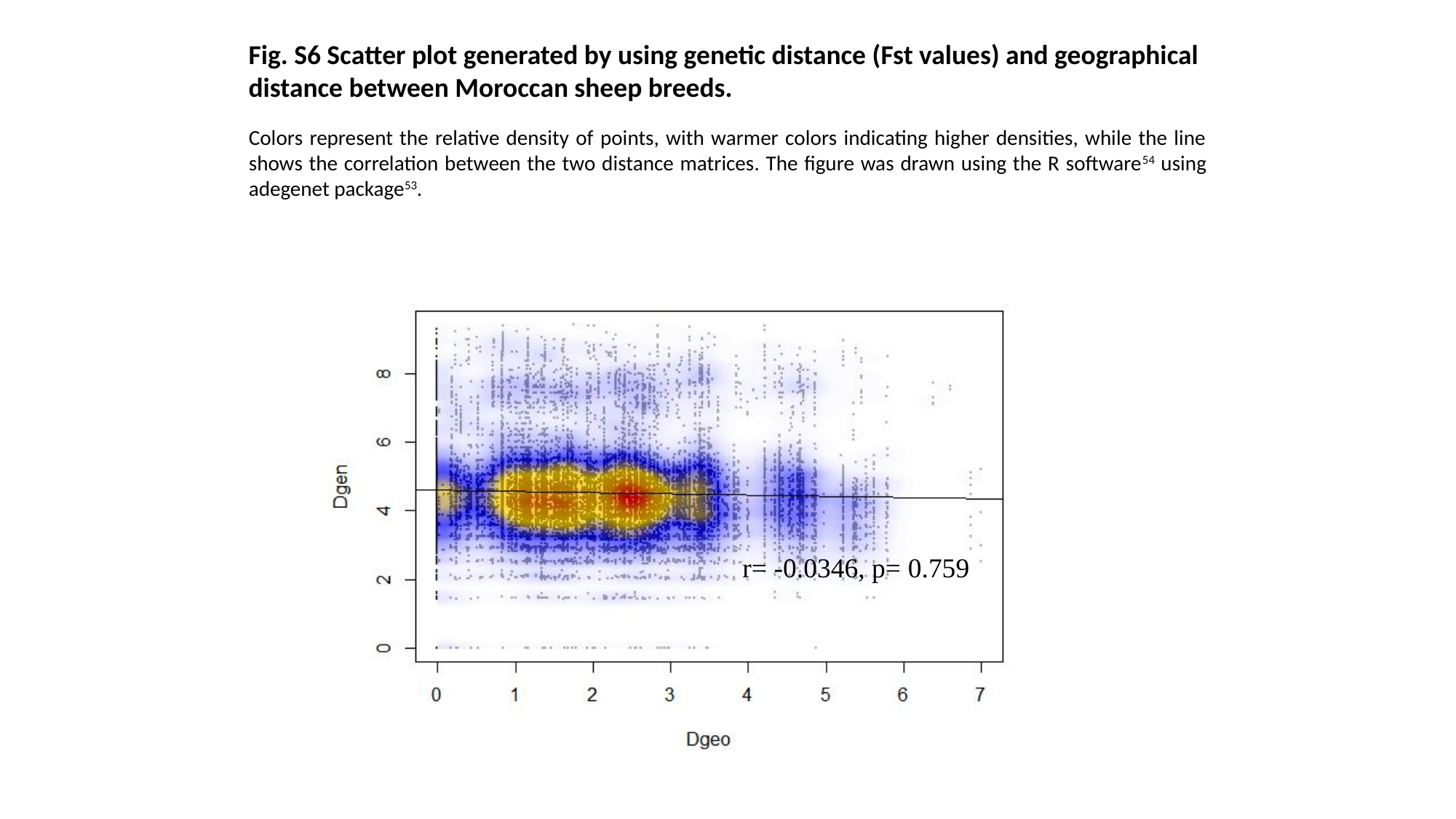

Fig. S6 Scatter plot generated by using genetic distance (Fst values) and geographical distance between Moroccan sheep breeds.
Colors represent the relative density of points, with warmer colors indicating higher densities, while the line shows the correlation between the two distance matrices. The figure was drawn using the R software54 using adegenet package53.
r= -0.0346, p= 0.759

## Slide 8
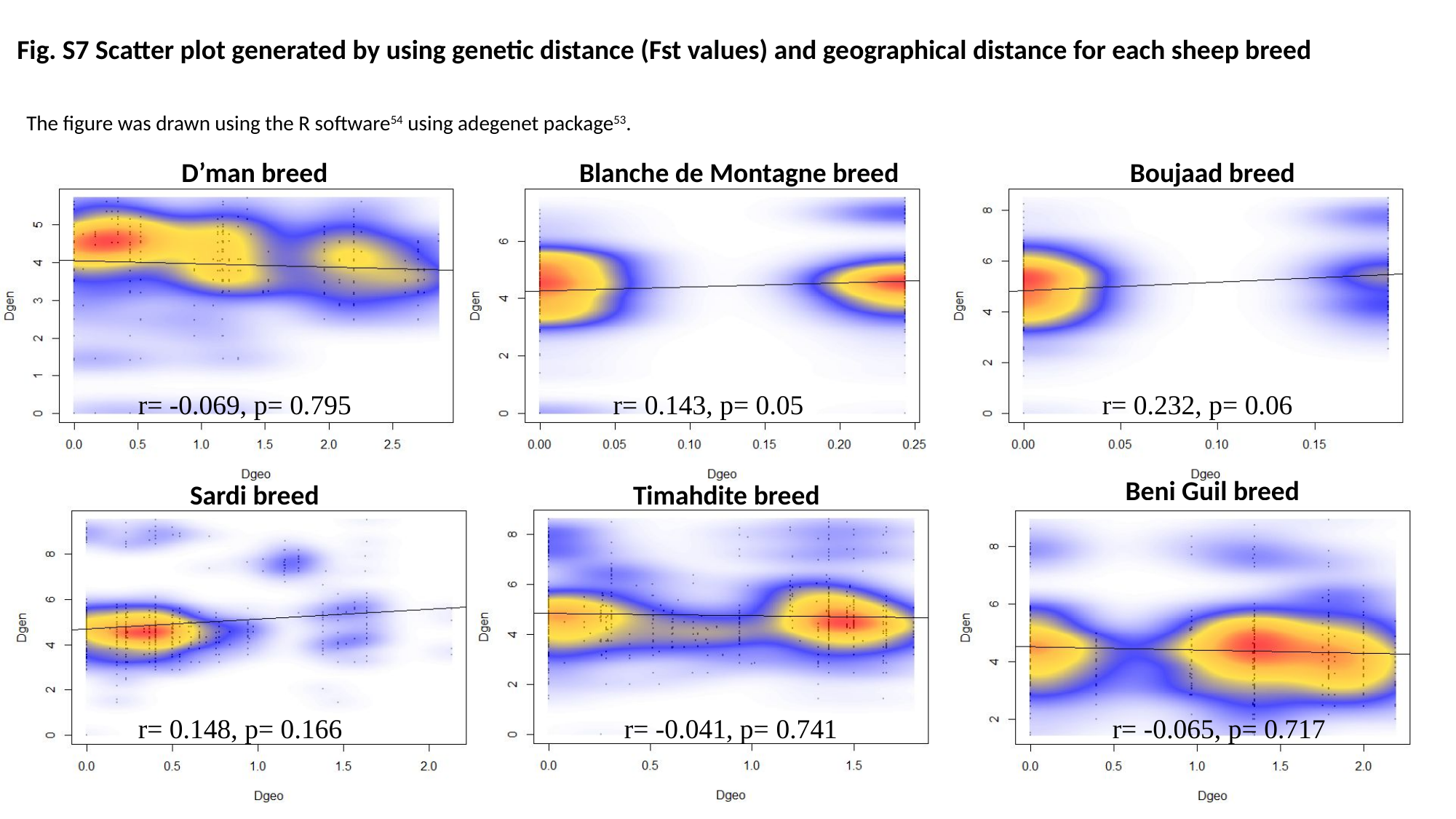

Fig. S7 Scatter plot generated by using genetic distance (Fst values) and geographical distance for each sheep breed
The figure was drawn using the R software54 using adegenet package53.
D’man breed
Blanche de Montagne breed
Boujaad breed
r= -0.069, p= 0.795
r= 0.143, p= 0.05
r= 0.232, p= 0.06
Beni Guil breed
Sardi breed
Timahdite breed
r= 0.148, p= 0.166
r= -0.041, p= 0.741
r= -0.065, p= 0.717
