# Supplementary material for: How have sheep breeds differentiated from each other in Morocco? Genetic structure and geographical distribution patterns
Source: Genet Sel Evol. 2021 Nov 4;53:83. doi: 10.1186/s12711-021-00679-2 (PMC8567669; doi:10.1186/s12711-021-00679-2)
Supplement: Supplementary file 3 — Additional file 3: Table S3. Analysis of molecular variance (AMOVA) in the six Moroccan sheep breeds. df: degree of freedom; SSD: sum of squares; MSD: mean squared deviations. Table S4. AMOVA results with different breed groupings. Table S5. Genetic diversity indices of subpopulations of the D’man, Sardi, and Timahdite breeds. [file 12711_2021_679_MOESM3_ESM.docx]

**TableS3** Analysis of molecular variance (AMOVA) in the six Moroccan sheep breeds.

d.f: Degree of freedom; SSD: Sum of squares; MSD: Mean squared deviations

| **Source of variation** | **d.f.** | **SSD** | **MSD** | **Variation components** | **Percentage of variation (%)** | **Fixation indices** | **P-values** |
| --- | --- | --- | --- | --- | --- | --- | --- |
| Between populations | 5 | 86.175 | 17.234 | 0.133 | 1.332 | F_SC_=0.0133 | 0.02 |
| Between samples within populations | 31 | 329.768 | 10.638 | 0.287 | 2.881 | F_IS_=0.0288 | 0.19 |
| Within samples | 154 | 1469.568 | 9.543 | 9.543 | 95.787 | F_IT_=0.0421 | 0.05 |
| Total | 190 | 1885.511 | 9.924 | 9.962 | 100.000 |  |  |

**Table S4:** AMOVA results of breed grouping in two sets

| **Groups** | **Source of variation** | **d.f.** | **SSD** | **MSD** | **Variation components** | **Percentage of variation (%)** | **Fixation indices** | **P-values** |
| --- | --- | --- | --- | --- | --- | --- | --- | --- |
| **(1)**  (Sardi, Boujaad, Timahdite, Beni Guil)  (D’man, Blanche de Montagne) | Between groups | 1 | 18.2630 | 18.2631 | 0.0156 | 0.1566 | F_CT_=0.0015 | 0.3 |
|  | Between populations within groups | 4 | 67.9116 | 16.9779 | 0.1246 | 1.2501 | F_SC_=0.0125 | 0.03 |
|  | Between samples within populations | 31 | 329.768 | 10.6377 | 0.2871 | 2.8792 | F_IS_= 0.0292 | 0.15 |
|  | Within samples | 154 | 1469.568 | 9.5427 | 9.5427 | 95.7141 | F_IT_=0.0428 | 0.02 |
|  | Total | 190 | 1885.5104 | 9.9237 | 9.9699 | 100.0000 |  |  |
| **(2)**  (Sardi, Boujaad, Timahdite)  (Beni Guil, D’man, Blanche de Montagne) | Between Groups | 1 | 21.8227 | 21.8227 | 0.0602 | 0.6024 | F_CT_=0.0060 | 0.15 |
|  | Between populations within groups | 4 | 64.3520 | 16.0880 | 0.0966 | 0.9675 | F_SC_=0.0097 | 0.10 |
|  | Between samples within populations | 31 | 329.7675 | 10.6377 | 0.2871 | 2.8744 | F_IS_= 0.0292 | 0.17 |
|  | Within samples | 154 | 1469.5682 | 9.5427 | 9.5427 | 95.5557 | F_IT_=0.0444 | 0.05 |
|  | Total | 190 | 1885.5104 | 9.9237 | 9.9865 | 100.000 |  |  |
| **(3)**  (Sardi, Boujaad, Timahdite, Blanche de Montagne)  (Beni Guil, D’man) | Between Groups | 1 | 26.9610 | 26.9611 | 0.1639 | 1.6316 | F_CT_=0.0163 | 0.01 |
|  | Between populations within groups | 4 | 59.2136 | 14.8034 | 0.0494 | 0.4913 | F_SC_=0.0049 | 0.20 |
|  | Between samples within populations | 31 | 329.7675 | 10.6377 | 0.2871 | 2.8583 | F_IS_= 0.0292 | 0.19 |
|  | Within samples | 154 | 1469.5682 | 9.5426 | 9.5426 | 95.0187 | F_IT_=0.0498 | 0.05 |
|  | Total | 190 | 1885.5104 | 9.9237 | 10.0429 | 100.0000 |  |  |
| **(4)**  (Sardi, Timahdite, Blanche de Montagne)  (Beni Guil, D’man) (Boujaad) | Between Groups | 2 | 38.9810 | 19.4905 | 0.0606 | 0.6071 | F_CT_=0.0061 | 0.2 |
|  | Between populations within groups | 3 | 47.1936 | 15.7312 | 0.0977 | 0.9787 | F_SC_=0.0098 | 0.12 |
|  | Between samples within populations | 31 | 332.2222 | 10.7168 | 0.2946 | 2.9523 | F_IS_=0.0299 | 0.18 |
|  | Within samples | 154 | 1467.1135 | 9.5267 | 9.5267 | 95.4619 | F_IT_=0.0454 | 0.04 |
|  | Total | 190 | 1885.5104 | 9.9237 | 9.9796 | 100.0000 |  |  |
| **(5)**  (Sardi)(Boujaad, Timahdite) (Blanche de Montagne)  (Beni Guil, D’man) | Between Groups | 3 | 64.2509 | 21.41696 | 0.2351 | 2.3527 | F_CT_=0.0235 | 0.03 |
|  | Between populations within groups | 2 | 21.9238 | 10.96188 | -0.0718 | -0.7183 | F_SC=_ -0.0074 | 0.48 |
|  | Between samples within populations | 30 | 315.2565 | 10.50855 | 0.2550 | 2.5517 | F_IS_=0.0259 | 0.15 |
|  | Within samples | 155 | 1484.0792 | 9.57471 | 9.5747 | 95.8139 | F_IT_=0.0419 | 0.02 |
|  | Total | 190 | 1885.5104 | 9.92374 | 9.9930 | 100.0000 |  |  |

**TableS5:** Genetic diversity indices of subpopulations of D’man, Sardi, and Timahdite breeds.

| Sub-populations | **N^a^** | **S^b^** | **P^c^** | **Sg^d^** | **Π^e^** | **sd (Pi)** | **H^f^** | **Hd^e^** | **sd (Hd)** |
| --- | --- | --- | --- | --- | --- | --- | --- | --- | --- |
| **D’man (Tafilalet)(mean value)** | 10 | 36 | 23 | 16 | 0.01595 | 0.00148 | 12 | 0.945 | 0.037 |
| **D’man (Draa/Dades)** | 10 | 20 | 11 | 9 | 0.01232 | 0.01740 | 10 | 1 | 0.045 |
| **Sardi** (**El Borouj and Kelaa Sraghna) (mean value)** | 14 | 63 | 31 | 32 | 0.02567 | 0.00579 | 13 | 0.993 | 0.030 |
| **Sardi (Beni Meskine)** | 14 | 38 | 15 | 23 | 0.01621 | 0.00179 | 14 | 1 | 0.027 |
| **Timahdite (Ghoualem)** | 18 | 61 | 31 | 30 | 0.02836 | 0.00486 | 18 | 1 | 0.019 |
| **Timahdite (Timahdite)** | 18 | 29 | 10 | 19 | 0.01100 | 0.00168 | 14 | 0.954 | 0.039 |

^a^Number of sequences (N), ^b^Segregated sites (S), ^c^Phylogenetically informative sites (P), ^d^Singletons (Sg), ^e^Nucleotide diversity (Π), Observed haplotypes (H), ^e^Haplotype diversity (Hd), sd: standard deviation.
